# Has Medicine Advanced?

**Published:** 1897-07

**Authors:** M. K. Lott

**Affiliations:** Cameron


### TEXAS MEDICAL JOURNAL.

ESTABLISHED JULY, 1885.

PUBLISHED MONTHLY.—SUBSCRIPTION $2.00 A YEAR.

VOL. XIII. AUSTIN, JULY, 1897. No. 1.

### Original Contributions.

For the Texas Medical Journal.

### HAS MEDICINE ADVANCED?

BY M. K. LOTT, M. D., CAMERON.

Read before the Brazos Valley Medical Association, Cameron, Texas.

GENTLEMEN:—Has medicine as a science advanced? Are the doctors of to-day at all superior to those of old?

The quiet fireside with Winslow's syrup upon the mantle and the busy mart chaffering over a gross of some universal cure-all, have asked and answered these questions a thousand times. But with such surroundings, the problem is forever unsolved. There is no patent right upon life and death. Science has no secrets; but, like the clear shining of a star, it is all light. Not then in the presence of patent nostrums, but in the hearing of scientific men and educated people, I shall try to contribute my share towards an answer to these questions. Let us deal fairly with each other and see who are right—those who doubt or those who believe—for the desire of the physician and the desire of the people must be one. The ends we aim at are the same, however widely we may differ as to methods, and methods must ultimately be the same, for I accept the theory of DeQuincy, that man is ever coming nearer to agreement, ever narrowing his differences, notwithstanding the interspace may cost an eternity to travel, for where the agreement is, and not where the difference is in the center of man's unities and not his diversities, there lies the magnetic center, toward which all thought that is potent, and all philosophy that is faithful, are eternally traveling—drifting toward a common center. That center in science, as well as morals, is truth, and truth is one.

There tends the drift, moved forward by the original order and persistent demand of man's ideal best. Not in anything yet is the outcome of the highest, but human thought is sleepless, and with its backward and its forward look conceives of treasures in advance as far richer than ours, as ours are richer than those of old. Holding to this as a central idea, I shall run rapidly over the history of medicine, giving a becoming prominence to a few of the master spirits who have signalized their generation, and thus bring the subject down to our own times, times which have culminated in a general fruitage more magnificent than the wildest fancies of the old-time philosopher ever dreamed. Having done this, it will require but little effort to show that the doctors of to-day are superior to those of old.

Medicine had its origin in the necessity of man's nature, and long antedates all written history. From the time that the sorrows of man and woman were multiplied, that thorns and thistles sprang from the fresh earth, and the sweat of the face became necessary to the eating of bread, we have all the concomitants necessary for human suffering; relief from suffering is the source from which it sprang. Pain is nature's cry for help; however imperfect the relief, the cry is still importunate. Whether it be the touch of the hand, the tone of the voice, the application of a leaf or a draught of cold water, it is human sympathy struggling with human suffering. From that struggle was born the art of medicine, and a spirit of benevolence was the presiding genius at its birth.

Totheor Hermes is said to have been the author of all science and all art; who he was, whether man or god or representative idea of philosophy, it is difficult now to determine. Writings claimed for him contained advice to kings, a dissertation upon the stars, the uses of astrology, and a treatise upon medicine, the authenticity of all of which is doubted, and doubted chiefly upon the ground that there is evinced a knowledge of anatomy more minute than is consistent with that early morning of the world. The earliest records we have of medicine are the Hebrew Scripture; while not professedly scientific, the Pentateuch is replete with hygienic instruction fully abreast with the latest writings upon that subject.

In the earlier times the sick and afflicted were placed by the wayside, in order that the passer-by might see and prescribe. Whether there was much wisdom in this or not, the patient had a multitude of counselors. In process of time these prescriptions became too numerous for unaided memory; they were then inscribed upon the walls of the temples and guarded with all the sacredness that attaches to a nation's archives. The priests having control of the temples, presently assumed exclusive control of the sick, but were required to practice in accordance with prescriptions on the walls. Any departure from them, if followed by death, was punished with death. The atrocity of the law was vindicated by the assumption, generally true, that the combined experience of many is superior to the judgment of one. This law, of course, was an obstacle mountain high to all progress in the healing art.

Hippocrates, who lived four hundred years before the Christian era, is denominated the father of medicine. His name stands out in bold relief, towering above all those of former ages. He announced the Vis Medicatrix Naturæ, a doctrine of which the world has never since lost sight. The enlightened physician everywhere recognizes the medicinal force of nature, makes his diagnosis and shapes his course by this. A very large class of diseases, measles, scarlatina, whooping-cough, small-pox, and so on, are self-limited; as long as they pursue their natural history, to use an apparent paradox, they are healthful diseases and do not require treatment. But you do not know they will pursue their natural history, hence you watch and wait, not to cure, but to see that they do not depart from their natural history. Yes, Hippocrates is the father of medicine. There are those who refuse to admit this claim, holding that he simply gathered together the streams of knowledge coming from many sources. And yet this modern critic is a citizen of these United States, from whose slopes trickle the thousands of streams that unite to form the father of waters. As well deny to the Mississippi the title of father of waters as to Hippocrates the title of father of medicine. In either case the streams from many sources presently run into one magnificent channel.

The next name that stands as a monument to mark the flight of time is that of Galen, in the second century of the Christian era. It is said of him that in thonght and in action he was free, and freedom of thought, aided by a clear understanding, retentive memory and indomitable energy made him master of all, anatomy, physiology, chemistry, materia medica, and therapeutics. All these we have enlarged and made more minute, but what was then known, Galen knew. If the student of today will rub away the accumulated mold of sixteen centuries, he will find much, that he read in yesterday's journal, inscribed upon the monument which Galen erected, which inscriptions are immortal, outlasting parchment or stone; for truth can never perish.

A fact in the history of many countries familiar to most of us is the existence of a lost river. Its waters dip for a space and disappear, yet at greater or less distance reappear with renewed freshness and redoubled volume. So in the middle or dark ages the stream of knowledge went out of sight, and left a blank in human progress for nearly one thousand years, yet recurring again with renewed freshness and redoubled volume with the revival of letters in the thirteenth century. Man relighted the torch by which hidden mysteries are sought, and with an energy heretofore unequaled, strode grandly forward in that pathway which has become the great highway of the nation, and points toward the secret place where truth sits—sits waiting to reward her worshipers with rich treasures, and waits until found and recognized by the last earnest searcher on the earth; for if truth is somewhere else, God is a myth and human life a failure. Imperial thought, ever restless, ever inquisitive, dares to be imperial. Knocking at the door of mysteries and compelling them to open to view. System after system has arisen, lived its day and declined; declined often in the limetime of him who laid its foundation, and yet each in its turn has enriched the great storehouse of knowledge, a real benefaction to man.

Hoffman, Cullen, Sylvius and Brown are names familiar to the medical historian, with their theories; and a little later another extravaganza flashed into life, bewildering the philosophystus, preying upon the gangling and out of tune nervous system, first of England, then of America, was the Hahnemanic —"similia simillibus curantur" system of homeopathy. Of all wild vagaries it is the wildest of the cobwebry; it is the most gossamer of all attenuations; it is the finest spun; it is the very delirium tremens of medical philosophy. Away with such transcendental folly.

In the sixteenth century, Ambrose Pare applied the ligature, and surgery received a rich treasure in its discovery.

The seventeen century is marked by the discovery of the circulation of the blood by Harvey. There had been glimmerings of light long anterior to this time. The theory of the older anatomists was that the veins were the blood vessels, that the arteries were air carriers; the passage of blood from the one to the other through the heart, thence to the lungs to be purified. All this was involved in inexplainable mystery until the great English Harvey, gathering up the rays of light of all the ages past, and adding them to the white flame of his own genius, laid bare forever the mechanism of the inward and the outward flow; and science saw and recognized the circulation of the blood.

The succeeding century, the eighteenth, is marked by the discovery of vaccination by Sir Edward Jenner. It is the molehill that has grown into a mountain. The molehill was ridiculed and scoffed at by our ancestors. Their descendants over the earth gaze with a feeling akin to worship upon the mountain. A single coral insect lying at the bottom of the sea is an object of unimportant interest in its silent, though ceaseless work, yet above its solitary mooring, in process of time, there rises a continent covered with verdure and beauty and life. So from the long unobserved pustule on the hand of the English milkmaid, Jenner built a system of thought that has preserved the beauty and vitality not only of a continent, but the world. All honor to Jenner!

The nineteenth century has been the crowning glory of the ages. Invention and discovery have chased one another down like the waves of the sea. Following in such rapid succession that even their mention is forbidden in a paper like this. Illustrious names that have lighted the earth, shine with undimned splendor from all parts of the world. Pasteur, Koch, Lister and their co-laborers standing in the very vanguard; and in the United States illustrious names illumine the land from Boston to Texas. Drake, Rush, Cobb, Wister, Gross, Sims, Stone and a host of others are household names throughout the land. The skill of the inventor has been added to the genius of the doctor in perfecting his knowledge and treatment of disease. We have the microscope with its delicate object lenses, bringing visibly before the eye the infusorial and bacterial world, and enabling Lister to establish the principles that bear his name, and are the parent of sepsis and antisepsis in surgery. We have the sphigmograph that writes in legible lines the varying pulse wave, and perchance reveals some abnormal heart. We have the fever thermometer that tells the temperature of the body morning, noon and night, and possibly explains some hidden source of irritation undiscoverable by touch or sight. We have the ophthalmoscope that exposes the mechanism of the eye, and brings palpably before the physician the mote or fibre that perverts or dims your vision. We have the laryngoscope that opens up the pathway of the human voice, and enables us to touch the very cord that is out of tune. We have the aspirator with its hollow needle and pump action, by which fluids in all parts of the body are quietly pumped out. The peri-cardium, the pleura, the peritoneum, the liver have all been reached with safety, even from the cranial cavity floods upon the brain have been removed. We have Esmarch's bloodless bandage. The surgeon of old, with white apron and rolled up sleeves, was a spectacle gastly and repellant enough even for strong masculine nerves. The surgeon of to-day takes off your arm or your leg, loses no drop of blood, does not soil his wristbands or stain the blade of his knife. We have the hypodermic syringe, through whose needle point we pour a drop of lethe into the very lap of pain, and hold in abeyance with absolute certainty the most terrible suffering. We have chloroform, that has uncrowned old king pain and given us the reign of that gentler spirit we call anæsthesia. We have harnessed the lightning and brought electricity to the aid of the physician and surgeon, and have made of it a willing aid to the alleviation and cure of diseases. Læennec listened through his cedar tube to the normal and diseased sounds of the lungs and the heart, and mapped out for us a field as untrodden as a native forest, and now we have the perfected stethoscope, enabling us to distinguish the slightest departure from the normal sound of the heart and lungs. And then we have the X ray, added by Prof. Roentgen in the recent past. With this wonderful instrument foreign substances situated in any part of the human body are photographed, and their position brought as visibly before the eye of the surgeon as the bee and the honey comb in its glass hive.

Antitoxine now challenges the attention and admiration of the scientific world. The reports of the wonderful results secured by their agents are encouraging. Before the sun rises on the twentieth century, consumption, diphtheria and allied diseases will have fallen before the antitoxine treatment, and will cease their fearful ravages.

We do not claim to have robbed old king death, but we do claim to have set back the hour hand of time, and to have added by a tithe the years allotted to man, and other years will be added as preventive medicine and sanitary science are better understood and practiced. We will postpone the "breaking of the golden bowl." We are living in the most enlightened age of the world; under a constitutional government that guards the rights of every fibre and tendril of the most complex civilization the world has ever seen. Autocracy is passing away, and a spirit of representative government is jostling the thrones of all the earth, advancement and progress in every department of human thought is unprecedented in the ages past. Bridge building has grown from fallen trees and rough slabs to the interlacing of wire that spans the Niagara and the Mississippi. Architecture has grown from mud walls roofed in by branches of trees to the splendid capitols of New York and Texas. Music has grown from the simple heart-note blown through hollow reeds to the orchestral renderings of Mozart, Handl and Bethoven. Who shall say, then, that medicine, the dearest of human interests, has not kept fully abreast of the times?

